# Longitudinal plasmode algorithms to evaluate statistical methods in realistic scenarios: an illustration applied to occupational epidemiology

**DOI:** 10.1186/s12874-023-02062-9

**Published:** 2023-10-18

**Authors:** Youssra Souli, Xavier Trudel, Awa Diop, Chantal Brisson, Denis Talbot

**Affiliations:** 1 Institute for Stochastics Johannes Kepler University, Linz, Austria; 2 Université Laval, Département de médecine sociale et préventive, Québec, Canada; 3 Centre de recherche du CHU de Québec - Université Laval, Axe santé des populations et pratiques optimales en santé, Québec, Canada

**Keywords:** Causal inference, Plasmode, Psychosocial stressors, Random forest, Regression modeling, Simulation, Longitudinal data

## Abstract

**Introduction:**

Plasmode simulations are a type of simulations that use real data to determine the synthetic data-generating equations. Such simulations thus allow evaluating statistical methods under realistic conditions. As far as we know, no plasmode algorithm has been proposed for simulating longitudinal data. In this paper, we propose a longitudinal plasmode framework to generate realistic data with both a time-varying exposure and time-varying covariates. This work was motivated by the objective of comparing different methods for estimating the causal effect of a cumulative exposure to psychosocial stressors at work over time.

**Methods:**

We developed two longitudinal plasmode algorithms: a parametric and a nonparametric algorithms. Data from the PROspective Québec (PROQ) Study on Work and Health were used as an input to generate data with the proposed plasmode algorithms. We evaluated the performance of multiple estimators of the parameters of marginal structural models (MSMs): inverse probability of treatment weighting, g-computation and targeted maximum likelihood estimation. These estimators were also compared to standard regression approaches with either adjustment for baseline covariates only or with adjustment for both baseline and time-varying covariates.

**Results:**

Standard regression methods were susceptible to yield biased estimates with confidence intervals having coverage probability lower than their nominal level. The bias was much lower and coverage of confidence intervals was much closer to the nominal level when considering MSMs. Among MSM estimators, g-computation overall produced the best results relative to bias, root mean squared error and coverage of confidence intervals. No method produced unbiased estimates with adequate coverage for all parameters in the more realistic nonparametric plasmode simulation.

**Conclusion:**

The proposed longitudinal plasmode algorithms can be important methodological tools for evaluating and comparing analytical methods in realistic simulation scenarios. To facilitate the use of these algorithms, we provide R functions on GitHub. We also recommend using MSMs when estimating the effect of cumulative exposure to psychosocial stressors at work.

## Introduction

Simulation studies are commonly used to examine and compare the performance of different statistical methods. Because multiple datasets can be randomly generated, they can limit the impact of random variability on their results. In addition, because data are generated from known mathematical equations, it is often possible to determine analytically the true value of the parameters of interest, thus allowing direct comparison between the estimates and the truth. However, synthetic simulations generally lack realism because their data are often generated based on arbitrary parameters. Thus, they cannot reflect the complexity of real-life data, raising concerns regarding the generalizability of their results. Alternatively, it is possible to compare different methods using real-life data. Although more realistic, the results of such studies can be difficult to interpret, since the differences (or lack of differences) may simply be due to random fluctuations. Moreover, in the absence of a “gold standard”, it is not possible to assess the bias of the estimators or the coverage of the confidence intervals.

Plasmode simulations were described by Vaughan et al. (2009) [1] as a more natural process to generate data and to account for the complex structure of real data. More specifically, plasmode refers to a general framework wherein simulated data are generated using a combination of information from real data and known mathematical equations. Franklin et al. (2004) [2] proposed an R package, Plasmode, to perform plasmode simulations for datasets with baseline covariates, a binary point-exposure, and a binary, continuous or time-to-event outcome. This simulation framework is becoming increasingly popular for evaluating statistical methods (e.g., [3–6]). To the best of our knowledge, there currently exists no plasmode algorithm for simulating both time-varying exposures and time-varying covariates. Such features are ubiquitous in longitudinal data. Simulating realistic longitudinal data can be challenging, notably because of the large amount of data-generating equations that needs to be specified.

In this article, we propose two longitudinal plasmode algorithms for generating data with time-varying exposure and covariates. These algorithms take a real dataset as an input and determine the data-generating equations by estimating the relations between the variables either using parametric models or nonparametric models. Both algorithms were devised in such a way that it is possible to estimate the true value of the parameter of interest using a Monte Carlo simulation. The benefits of these algorithms are twofold. First, they alleviate the need to manually specify multiple data-generating equations since these equations are determined from the observed data. Second, for the same reason, the generated data are expected to be more similar to the real data than what could realistically be achieved using conventional simulation approaches. As such, these algorithms allow comparing statistical methods for analyzing longitudinal data in realistic scenarios, while benefiting from the ability to generate multiple simulated datasets and thus control the Monte Carlo error.

The motivation for developing these longitudinal plasmode algorithms was to compare different confounding adjustment methods when estimating the effect of cumulative exposure over time to psychosocial stressors at work (PSW) on blood pressure. Multiple studies have investigated such effects, often using traditional adjustment methods where models are either adjusted for baseline covariates only or for both baseline and time-varying covariates [7]. Such adjustment methods may be inappropriate if some covariates have a double role of confounders and mediators, since adjustment for time-varying confounders leads to an overadjustment bias, and not adjusting for these variables leads to residual confounding bias. It has been argued that this exposure-confounder feedback could be present in the PSW context [8]. Marginal structural models (MSMs) are well-known methods to estimate the effect of time-varying exposures while controlling for time-varying covariates [9]. However, these models have only scarcely been used in the context of PSW (for example [8, 10–13]). Various estimators of the parameters of MSMs have been proposed. Some are relatively simple to implement, such as inverse probability of treatment weighting (IPTW), and others are more complex but have more desirable theoretical properties, such as targeted maximum likelihood estimation (TMLE). Advantages of TMLE include that it is expected to have a smaller variance than IPTW, and that it can naturally be combined with machine learning algorithms that reduce the amount of statistical assumptions required for consistent estimation. While TMLE combined with machine learning is theoretically superior to traditional methods, whether the benefits are worth the additional analytical complexity in real data analyses may vary between fields of application. Multiple studies have compared MSMs with traditional approaches in real data analyses [14]. While both traditional approaches and MSMs yield similar results in multiple situations, the effect estimates were found to differ substantially in others and even lead to opposing conclusions in a few cases [14]. As such, empirical comparisons of MSMs with traditional approaches in area-specific settings are important to guide analysts. Our proposed plasmode algorithms were originally developed to compare traditional methods with estimators of the parameters of an MSM in the context of PSW effect estimation. However, these algorithms are general and can be used in other fields to carry out simulation studies on longitudinal data with time-varying exposure and covariates.

In the remainder, we first provide a motivating example where we estimate the effect of cumulative exposure to PSW on blood pressure using different methods. We then describe plasmode simulations for a point-exposure, before presenting the two longitudinal plasmode algorithms we have developed, one which is parametric and another which is nonparametric. A simulation study based on these algorithms is then presented. We end the paper with some recommendations.

## Motivating example

### Data

The PROspective Québec (PROQ) Study on Work and Health is a cohort study of 9,189 white-collar workers aged 18 to 65 years at baseline from 19 public or semi-public organizations in Québec, Canada [7]. A nested cohort comprising 2,200 workers from 3 public insurance institutions was initiated in 2000-2004 with follow-ups after 3 (2004-2006) and 5 years (2006-2009). The participation rate was 80.1% at baseline and 85% at years 3 and 5. The data from this nested cohort study are used in the current analysis. Participants completed self-reported questionnaires on work characteristics and risk factors for ambulatory blood pressure (ABP). Body weight and height were recorded by a trained nurse to calculate body mass index ($kg/m^2$). The exposure (PSW) was measured at baseline and both follow-ups according to a well-known theoretical model, the effort-reward imbalance (ERI) model. This model postulates that an imbalance between the efforts expended in the work environment and the rewards received in return has deleterious consequences on health [15]. Efforts and rewards were measured by means of a validated questionnaire [16]. The ERI ratio was obtained by dividing the effort score by the reward score. This ERI ratio was dichotomized, such that a ratio greater than 1 indicates exposure to PSW (PSW = 1, otherwise PSW = 0) [15]. Then, the cumulative PSW was classified into five categories: never exposed (0, 0, 0), intermittent exposure (0, 1, 0 or 1, 0, 1), exposure that ceased during the follow-up (1, 0, 0 or 1, 1, 0), onset exposure (0, 1, 1 or 0, 0, 1) or chronic exposure (1, 1, 1). The outcome for the present study was ABP measured at the end of the follow-up (2006-2009). ABP was measured at the participants’ workplace using the oscillometric device Spacelabs 90207 [17]. Potential confounders included gender, age at baseline, level of education (less than college, college completed, university completed), smoking (current or non-smoker), alcohol use ($<1$ drink/week, $1-5$ drinks/week, $\ge 6$ drinks/week), family history of cardiovascular disease (yes or no), sedentary lifestyle (physical activity <1/week or $\ge 1$/week). Finally, body weight and height were measured by a trained nurse to calculate body mass index ($<18.5$, $18.5-25$, $\ge 25 kg/m^2$). These covariates were selected a priori because they affect blood pressure [18, 19] and may also be associated with exposure [20]. Covariates were time-varying except for gender, age at baseline and education. The data were anonymized by removing information identifying the patient prior to analysis.

To simplify the illustration, only subjects with complete data for the aforementioned covariates, exposure and outcome were considered. Women who were pregnant at the last follow-up were also excluded because of the impact of pregnancy on blood pressure. Finally, workers that were working less than 21 hours per week at any time point were excluded. The final sample consisted of 1,576 workers, of whom 925 were women and 651 were men.

### Marginal structural models and notation

**Fig. 1:**
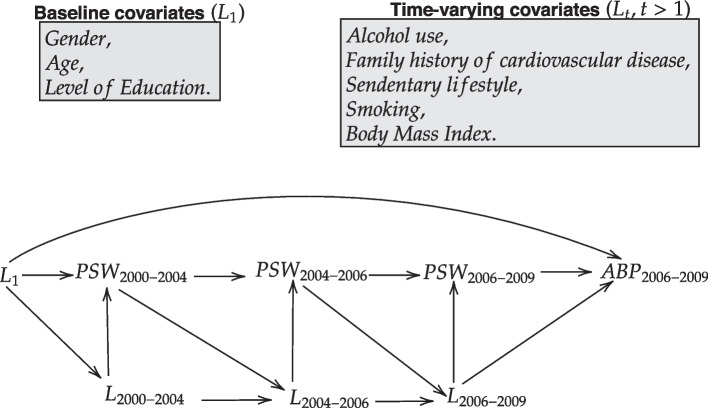
Directed acyclic graph representing the presumed relations between the exposure (psychosocial stressors at work; PSW), the outcome (ambulatory blood pressure; ABP) and the covariates. Some arrows between the time-varying exposure, the time-varying covariates and the outcome were omitted to simplify the presentation

The objective of our motivational illustration was to estimate the effect of cumulative exposure to ERI on ABP at the end of the follow-up. As mentioned in the introduction, the use of MSMs is theoretically justified by the possible presence of exposure-confounder feedback (see Fig. 1).

We now introduce some notation in order to formally present MSMs and their estimators. Let *K* be the number of follow-up time points, $i = 1, ..., n$ the observations, $L_t$ the covariates at time *t* ($t = 1, ..., K$), $A_t$ the exposure at time *t*, and *Y* the outcome at the end of the follow-up. We assume the following time-ordering of the variables ($L_1, A_1, ..., L_K, A_K, Y$). We use an overbar to denote the history of a variable up to and including a given time, for example, $\bar{A}_4 = \{A_1, A_2, A_3, A_4\}$. As a notational shortcut, the index is dropped to represent the complete history of a variable (for example, $\bar{A} \equiv \bar{A}_K$). The counterfactual outcome $Y^{\bar{a}}$ is the value that the outcome would have taken if, possibly contrary to fact, the exposure had been $\bar{a}$. Using this notation, an MSM can be formally defined as a model for the counterfactual outcome according to the exposure history:

1 $$\begin{aligned} \mathbb{E}(Y^{\bar{a}})=f(\bar{a}), \end{aligned}$$

where *f*() is some regression function. In our analysis, we assume $f(\bar{a}) = \gamma _0 + \gamma _1 intermittent + \gamma _2 cessation + \gamma _3 onset + \gamma _4 chronic$, where *intermittent*, *cessation*, *onset* and *chronic* are dummy variables for intermittent, ceased, onset and chronic exposure as defined previously, respectively.

Three well-known estimators of the parameters of an MSM are IPTW, g-computation and TMLE. These estimators require the following usual causal assumptions (see [9]): 1) $Y^{\bar{a}} \coprod A_t | \bar{A}_{t-1}, \bar{L}_t$ for all *t*, 2) $0< P(A_t = 1|\bar{A}_{t-1}, \bar{L}_{t}) < 1$, and 3) $\bar{A} = \bar{a} \Rightarrow Y = Y^{\bar{a}}$. The first assumption means there are no unmeasured confounders between the exposure at any time point and the outcome. Assumption 2 implies that each individual had nonzero probabilities of being exposed and of being unexposed at each time point. The final assumption means that the observed outcome corresponds to the counterfactual outcome under the observed exposure history.

The most popular estimation approach of an MSM is IPTW. The IPTW entails fitting a weighted regression of the observed outcome on the observed exposure history. The stabilized weights are derived from the probability of exposure conditional on the covariates:

2 $$\begin{aligned} sw_{i}=\prod _{t=1}^{K}\dfrac{P(A_t=a_{t,i}|\bar{A}_{t-1}=\bar{a}_{t-1,i})}{P(A_t=a_{t,i}|\bar{A}_{t-1}=\bar{a}_{t-1,i},\bar{L}_t=\bar{l}_{t,i})}. \end{aligned}$$

Intuitively, these weights create a pseudo-population where the exposure at each time point is independent of previously measured confounders, thus mimicking a randomized trial relative to those confounders. G-computation is a generalization of the standardization approach. An algorithm for implementing the g-computation estimator of $\mathbb {E}[Y^{\bar{a}}]$ proceeds as follows:

1. Fit a regression of the outcome according to the exposure and covariate history $\mathbb {E}[Y|\bar{A}, \bar{L}]$.
2. For each observation, compute the predicted value under $\bar{A} = \bar{a}$ and $\bar{L} = \bar{l}_i$ and denote the result by $Q_{K, i}^{\bar{a}}$.
3. Recursively for $$t = K-1, ..., 1$$
  3.1. Fit a regression $$\mathbb {E}[Q_{t+1}^{\bar{a}}|\bar{A}_t, \bar{L}_t]$$
  3.2. Compute the predicted value $Q_{t, i}^{\bar{a}} = \hat{\mathbb {E}}[Q_{t+1}^{\bar{a}}|\bar{A}_t = \bar{a}_t, \bar{L}_t = \bar{l}_{t, i}]$
4. $$\hat{\mathbb {E}}[Y^{\bar{a}}] = \frac{1}{n}\sum _{i=1}^n Q_{1, i}^{\bar{a}}$$.

After all counterfactual means $\mathbb {E}[Y^{\bar{a}}]$ have been estimated, the parameters of the MSM can be estimated by regressing $\hat{\mathbb {E}}[Y^{\bar{a}}]$ on $\bar{a}$ as in Eq. (1). Alternative algorithms for implementing g-computation have been described and compared [21].

TMLE is closely related to IPTW and g-computation [22, 23]. Indeed, an algorithm for obtaining a TMLE of $\mathbb {E}[Y^{\bar{a}}]$ when *Y* is continuous is the same as the g-computation algorithm described previously, except that each $Q_{t, i}^{\bar{a}}$ is first updated (or fluctuated) before moving on to the next step:

$$\begin{aligned} Q_{t, i}^{\bar{a}, 1} = Q_{t, i}^{\bar{a}} + \varepsilon H_t(\bar{A}, \bar{L})_{t-1}, \end{aligned}$$

where

$$\begin{aligned} H_t(\bar{A}, \bar{L})_{t-1} = \frac{I(\bar{A}_{t-1} = \bar{a}_{t-1})}{\prod _{s=0}^{t-1}P(A_s|\bar{A}_{s-1}, \bar{L}_s)}, \end{aligned}$$

*I* is the usual indicator function, and $\varepsilon$ can be estimated as the sole coefficient of regression of *Y* on $H_t(\bar{A}, \bar{L})_{t-1}$ with $Q_{t, i}^{\bar{a}}$ as an offset term.

While all these estimators depend on the same causal assumptions, they depend on different statistical assumptions. IPTW requires modeling the exposure at each time point and thus additionally depends on correct statistical models for these components to yield consistent estimates. Similarly, g-computation requires the correct modeling of the outcome. On the other hand, TMLE uses both models for the outcome and for the exposure. TMLE is a doubly robust method that is consistent as long as either the exposure model or the outcome model is correctly specified [22, 24]. In addition, TMLE better combines with machine learning than either IPTW or g-computation from a theoretical point of view. Indeed, IPTW and g-computation require that their component model (exposure model or outcome model, respectively) is $\sqrt{n}$-consistent to achieve the $\sqrt{n}$-consistency that most usual statistical estimators have. On the other hand, TMLE can achieve $\sqrt{n}$-consistency under less stringent conditions, for example, if both the exposure and outcome models are estimated with $n^{1/4}$-consistent estimators [25, 26]. Many flexible machine learning algorithms have convergence rates that are lower than $\sqrt{n}$. As such, TMLE can be combined with more flexible machine learning algorithms than IPTW and g-computation while retaining desirable statistical properties. In particular, Super Learner, an algorithm that finds the best linear combination of multiple machine learning models minimizing the cross-validated risk, can be combined with TMLE to improve its performance [27, 28]. The Super Learner is also implemented and available in the R software. It is noteworthy that while TMLE better combines with machine learning methods than traditional approaches, several recent studies warn that including machine learning algorithms that are too flexible in the Super Learner when using TMLE or other double robust approaches can lead to invalid inferences [29, 30].

### Analysis

We estimated the effect of cumulative exposure to PSW using an MSM whose parameters were estimated using IPTW, g-computation, TMLE and TMLE with Super Learner (TMLE-SL). For IPTW and TMLE, the exposure models were logistic regression models whereas the outcome models for g-computation and TMLE were linear regression models, both including main terms only. For TMLE-SL, we used both generalized linear models and generalized additive models (GAM) for both the exposure and the outcome. We also considered standard regression approaches where ABP was modeled according to cumulative exposure with either adjustment for baseline covariates only or with adjustment for both baseline and time-varying covariates. All these approaches were implemented in the R software.

Standard errors for g-computation and TMLE were estimated using the nonparametric bootstrap method with 1000 replicates. For these methods, confidence intervals were calculated using the $2.5^{th}$ and $97.5^{th}$ quantiles of the bootstrap estimates. For TMLE-SL, the standard errors were estimated by computing the square-root of the variance of the empirical efficient influence curve divided by *n* [31]. For each method, we present the parameter estimates for each exposure levels (estimated ABP mean difference of intermittent, cessation, onset and chronic exposure vs never exposed), 95% confidence intervals (CI) and the computation time in seconds (s).

### Results

**Fig. 2:**
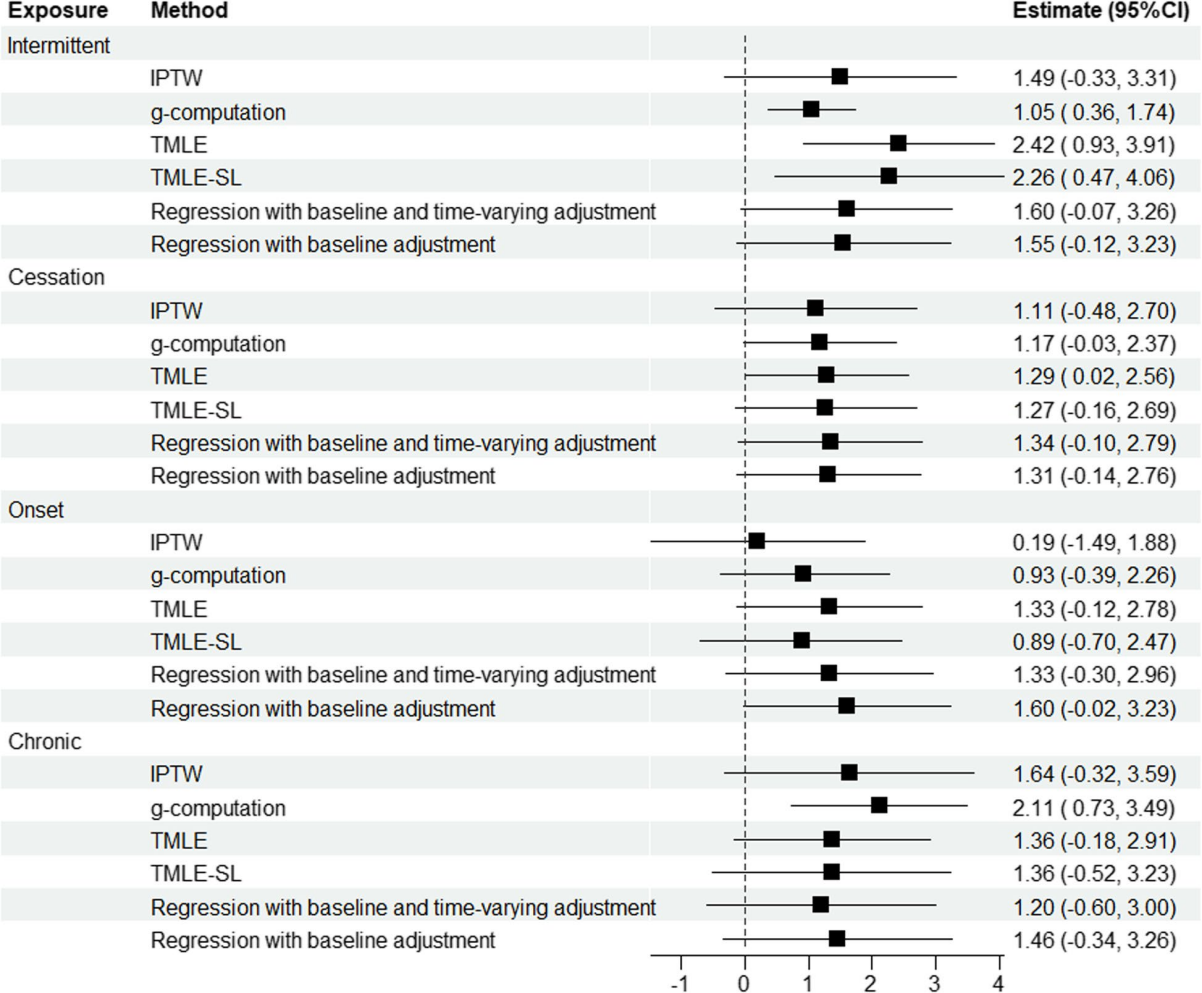
Estimated causal effects of categories of cumulative exposure to psychosocial stressors at work on ambulatory blood pressure (in mm Hg) using different estimation methods

Figure 2 presents the results of the data analysis. Overall, estimates differed somewhat importantly between estimation approaches. For example, the estimated ABP mean difference between onset exposure and never exposed was 0.19 mm Hg (CI: -1.49, 1.88) when using IPTW and 1.60 mm Hg (CI: -0.02, 3.23) when using a standard regression adjusted for baseline covariates only. As another example, the estimated ABP mean difference between intermittent and never exposed varied from 1.05 mm Hg (CI: 0.36, 1.74) with g-computation, to 2.42 mm Hg (CI: 0.93, 3.91) with TMLE. The width of the CIs also varied substantially between estimation methods, with g-computation yielding the narrowest CIs overall. Finally, the computation time was negligible (<1 second) for IPTW and classical approaches, whereas it was of 3.39 seconds for TMLE-SL, and 16-25 seconds for g-computation and TMLE with bootstrap. While these results showcase how different methods can produce different results when analyzing the same data, they do not permit concluding which performed best. Simulation studies, especially plasmode simulations, provide valuable additional information in that regard.

## Description of the plasmode algorithms

### Plasmode algorithm for single time point exposure

**Fig. 3:**
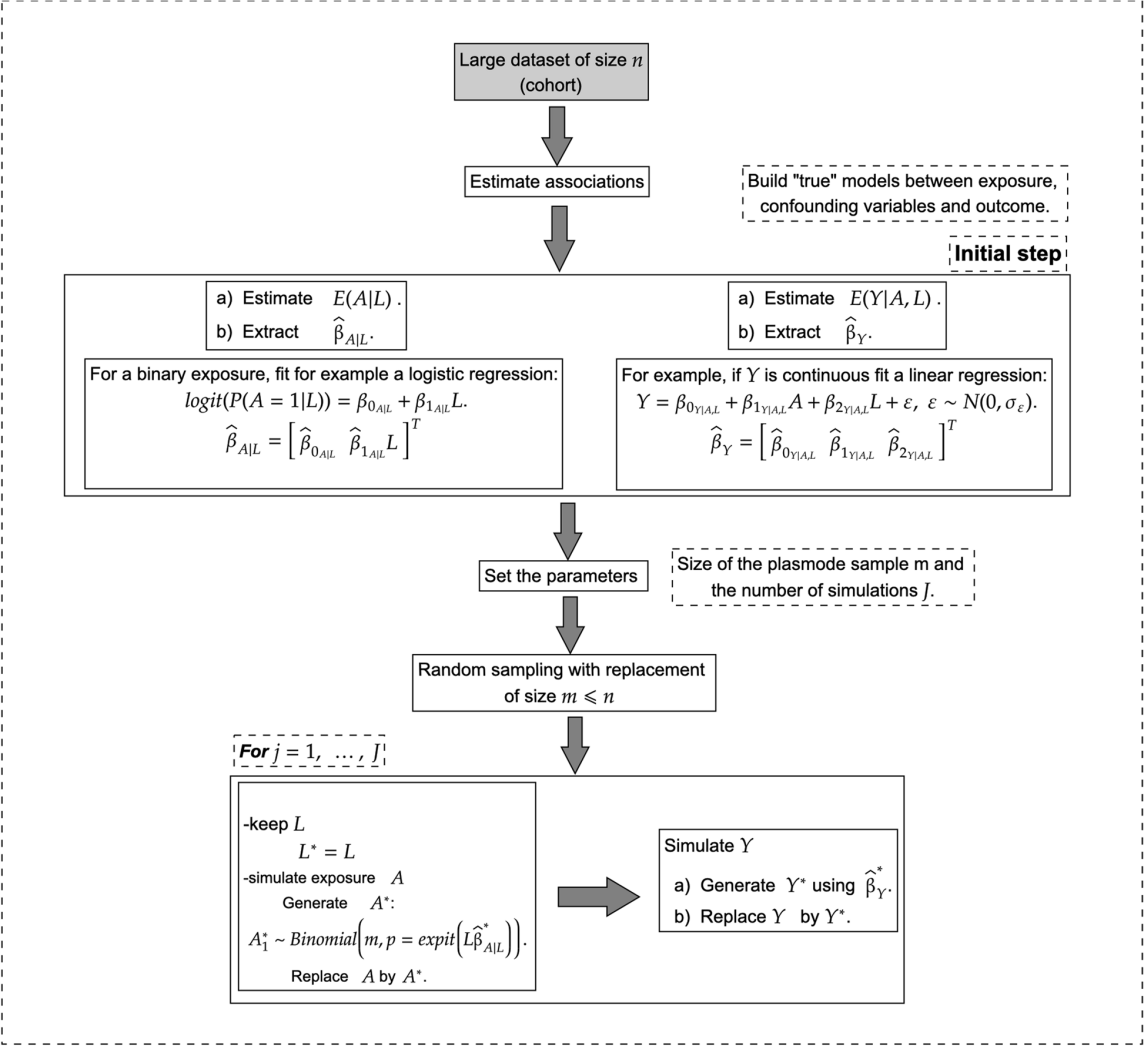
Visual representation of a plasmode algorithm for generating simulated data with a single time point exposure

Before introducing our proposed longitudinal plasmode algorithms, we first describe a typical plasmode algorithm for generating simulated data with an exposure measured at a single time point (see Fig. 3). Such an algorithm could be used to compare estimators of the effect of the exposure on the outcome, for example. We use the notation introduced in the previous section, but drop the time index since it is not needed. First, a “true” outcome model is fitted on the complete original dataset. For example, if *Y* is a continuous variable, the model could be a linear regression of *Y* on *A* and *L* with normal errors [2, 32]. Then, $m < n$ observations are sampled with replacement from the original dataset to form the basis of the plasmode data. The observed outcome is discarded and replaced by a synthetic outcome which is generated using the output of the true outcome model that was fitted earlier, and the sampled data on *A* and *L*. The resulting dataset comprising the sampled *A* and *L*, and the synthetically derived *Y* form the plasmode dataset. Because *Y* was generated using a known model, the true effect of *A* on *Y* can either be calculated analytically from the data-generating equations or by Monte Carlo simulation of counterfactual outcomes (for example, see [2]). Multiple plasmode datasets would typically be generated to reduce the Monte Carlo error (i.e., random fluctuations). As previously noted, this is only an example of a plasmode algorithm; other algorithms are possible. For example, instead of using resampled values of the exposure, *A* could be synthetically generated using a true exposure model that would be determined by fitting a model for the exposure according to baseline covariates on the complete original dataset.

### Parametric plasmode algorithm for time-varying exposure and covariates

The first proposed plasmode algorithm for time-varying exposure and covariates is a natural extension of the parametric plasmode algorithm for point-exposure described previously. This algorithm uses parametric regression models, whose parameters are determined from the original dataset, to generate the plasmode data. Because the causal effect of the cumulative exposure on the outcome depends on the exposure $\rightarrow$ confounders $\rightarrow$ outcome pathways, it is necessary to have known data-generating equations for most of the time-varying exposures and confounders, in addition to the outcome, to be able to determine the true effect. Both this parametric algorithm and the nonparametric algorithm described in the next subsection share the same rationale. First, it can be noted that the joint distribution of the data can be factorized as a product of conditional distributions:

$$\begin{aligned} f(\bar{A}, \bar{L}, Y) = f(Y|\bar{A}, \bar{L}) \prod _{t=1}^K f(L_t|\bar{A}_{t-1}, \bar{L}_{t-1})f(A_t|\bar{A}_{t-1}, \bar{L}_{t}), \end{aligned}$$

where any variable with a 0 index should be disregarded for notational convenience. Both algorithms entail estimating each of these conditional distributions using either a parametric or a nonparametric model. Then, data are sequentially simulated from the estimated conditional distributions. Based on the previous factorization, the algorithms thus allow simulating data from the estimated joint distribution of the data.

The first step of the algorithm consists in building “true” parametric models for generating the simulated exposure, confounders and outcome. To achieve this, the following models must be fitted to the original dataset: 1) $A_t$ conditional on ($\bar{A}_{t-1}$, $\bar{L}_{t}$) for $t = 1, ..., K$, 2) $L_t$ conditional on ($\bar{A}_{t-1}$, $\bar{L}_{t-1}$) for $t = 2, ..., K$, 3) *Y* conditional on ($\bar{A}$, $\bar{L}$). For example, continuous variables could be modeled using linear regression models with normal errors, binary variables could be modeled with logistic regression models, count variables can be modeled using Poisson regression models and a time-to-event outcome could be modeled using an accelerated failure time model. Denote by $\hat{\beta }_{A_t}$, $\hat{\beta }_{L_{t}}$ and $\hat{\beta }_Y$ the coefficients of the models for the exposure at time *t*, covariates at time *t* and outcome, respectively. Note that it is not necessary to include all previous variables as independent variables in each model. To simplify the model specification, a relevant subset could be chosen based on substantive or expert knowledge, or variables to be used could be randomly sampled (see for example [33]). Employing more parsimonious models may be essential when the number of available variables is large.

To generate a plasmode dataset, *m* observations are randomly sampled with replacement from the original dataset, but only $L_1$ variables are kept; all other variables are discarded. Then, the output of the “true” models (i.e., $\hat{\beta }_{A_t}$, $\hat{\beta }_{L_{t}}$ and $\hat{\beta }_Y$) are used to randomly generate synthetic data sequentially for $A_1$, $L_2$, $A_2$, ..., *Y*.

**Fig. 4:**
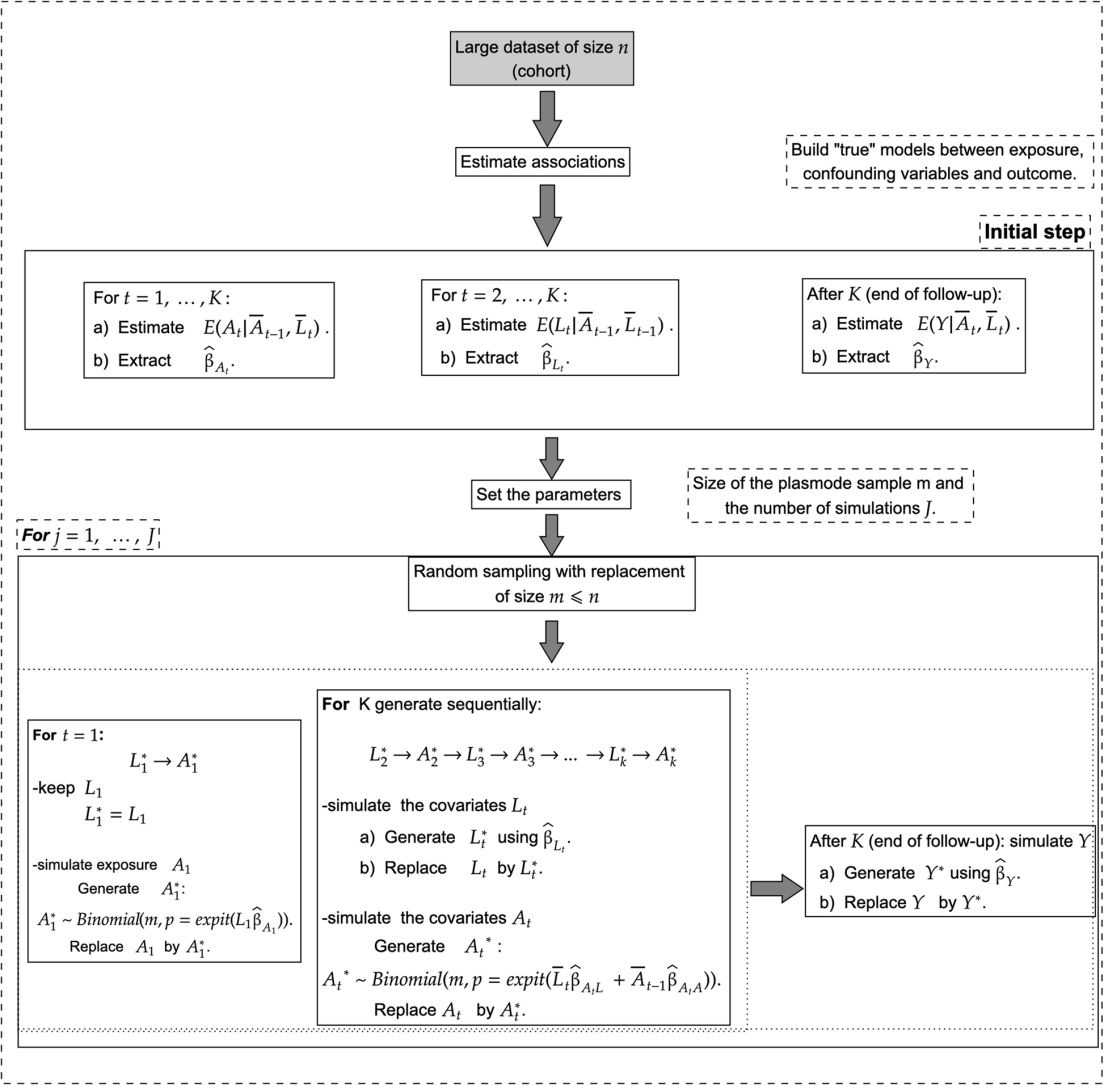
Visual representation of the proposed parametric plasmode algorithm for generating simulated data with time-varying exposures and covariates

A total of *J* plasmode datasets are generated using the algorithm. Figure 4 summarizes this parametric plasmode algorithm. The true effect can either be determined analytically from the data-generating equations or using Monte Carlo simulations. An algorithm for estimating the true parameters using Monte Carlo simulations in the specific example where causal effects are of interest is described in the “Counterfactual plasmode algorithms” section.

### Nonparametric plasmode algorithm for time-varying exposure and covariates

One disadvantage of the parametric plasmode algorithm we have described is that it uses parametric models to generate most of the data, whereas plasmode simulations should be designed to be as similar as possible to the real data. Parametric models may lack the required flexibility to achieve this goal. However, it is essential to have known data-generating equations to calculate the true values of the parameters. The nonparametric plasmode we propose uses random forest models to generate the data. Such models allow having known data-generating equations that are nonparametric and thus more similar to the real data. Random forest models are particularly useful when dealing with non-linear modeling and they run efficiently on large datasets which makes it a suitable choice for our nonparametric plasmode algorithm [34]. Extensions of random forest for time-to-event data are also available [35].

**Fig. 5:**
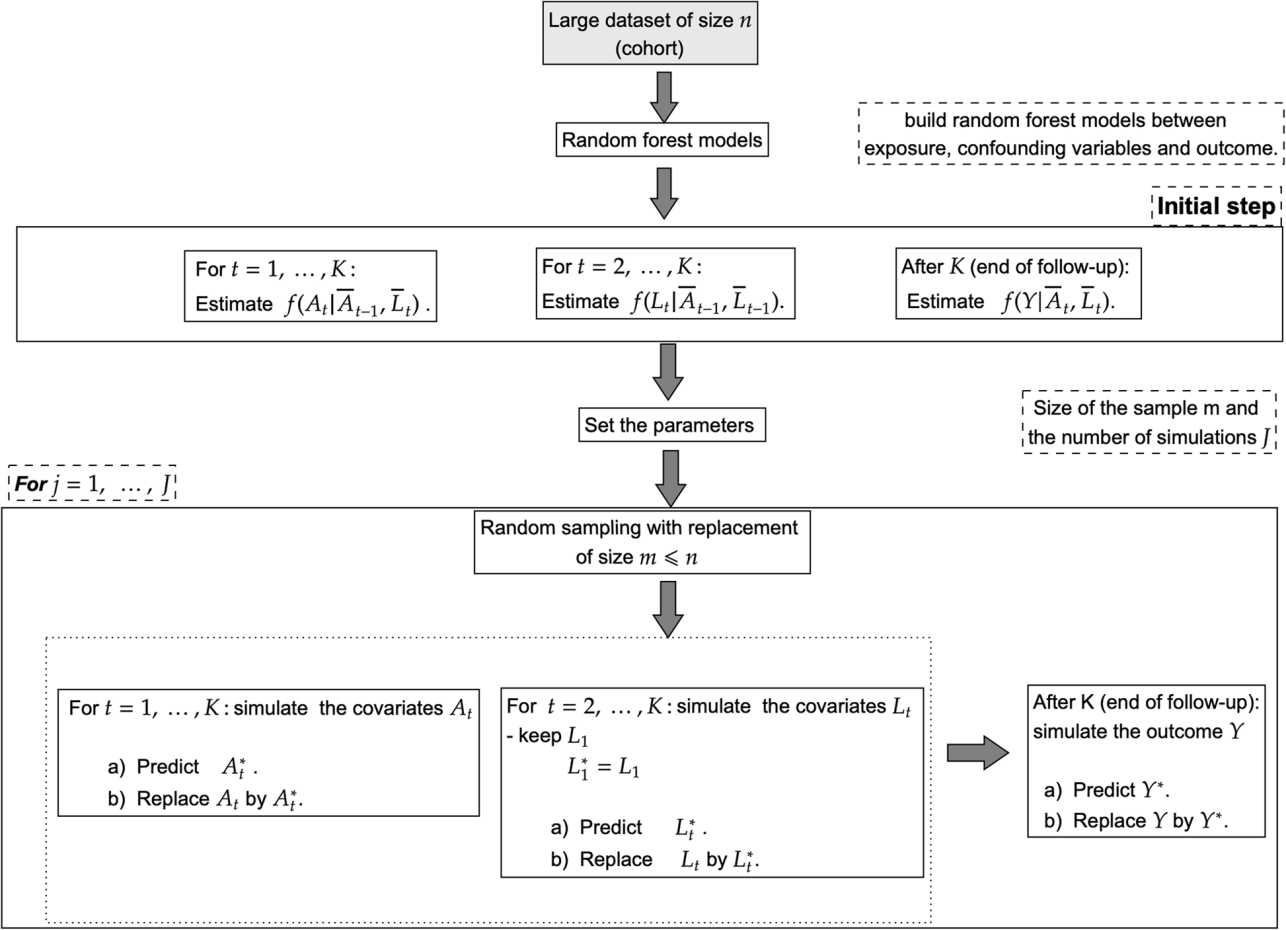
Visual representation of the proposed nonparametric plasmode algorithm for generating simulated data with time-varying exposures and covariates

Similar to the parametric plasmode, the nonparametric plasmode algorithm first entails fitting the following random forest models to the original data: 1) $A_t$ conditional on ($\bar{A}_{t-1}$, $\bar{L}_{t}$) for $t = 2, ..., K$, 2) $L_t$ conditional on ($\bar{A}_{t-1}$, $\bar{L}_{t-1}$) for $t = 2, ..., K$, 3) *Y* conditional on ($\bar{A}$, $\bar{L}$). Next, to generate a plasmode dataset, $m<n$ observations are randomly sampled with replacement from the original dataset. Only data on $L_1$ are kept; however, it is also possible to retain $A_1$. These data and the random forest models are then used to randomly generate synthetic values for $L_2$, $A_2$, ..., *Y* (see Fig. 5). One challenge with this nonparametric plasmode algorithm is that it is difficult to determine the true values of the parameters analytically, given the nonparametric nature of the data-generating models. Instead, Monte Carlo simulations can be used to estimate the true parameters.

### Counterfactual plasmode algorithms

We now describe a counterfactual plasmode algorithm that can be applied to determine the true values of the causal effect when generating data using either of our longitudinal plasmode algorithms. The general principle is to generate new datasets based on the same equations used in the plasmode algorithms, except that the exposure is deterministic instead of being random. More specifically, consider a given exposure pattern $\bar{a}$. Then, for each observation, simulated data on $L_2$, ..., $L_K$ are sequentially generated using the “true” confounder models, previously generated covariates and the fixed previous exposures. Finally, the counterfactual outcome $Y^{\bar{a}}$ is simulated using the “true” outcome model. This process is repeated for each possible exposure patterns, resulting in a dataset of $n \times 2^K$ observations. The true parameters of the MSM are then estimated by running a regression of the simulated counterfactual outcomes on the cumulative exposure. In simulation studies where the parameter of interest is not a causal contrast, a different algorithm for estimating the true values using Monte Carlo simulations may need to be devised.

### Practical implementation

We provide four R functions on GitHub (https://github.com/detal9/LongitudinalPlasmode) that implement the parametric and nonparametric plasmode algorithms as well as their related counterfactual algorithms. We briefly describe the use of these functions in this section.

First, some data preprocessing may be required. For example, because our functions do not support missing data, subjects with missing information either need to be excluded or their missing data need to be imputed. Because the algorithm does not take into account uncertainty, a single maximum likelihood imputation of the data may be deemed to be sufficient. Some levels of categorical variables may also need to be collapsed together to avoid problems when fitting the models. Categorical variables should be coded as factors in R. Note that the functions we provide currently support only continuous or categorical (binary or multinomial) covariates and binary exposures.

The functions all share similar arguments:

- data: A dataframe containing the real data to be used to generate simulated datasets.
- n: The sample size of the orignal data.
- nsmin: The number of simulated datasets to generate.
- timeobs: The number of time points.
- Y: The name of the outcome variable.
- id: The name of the variable representing the unique subject identifier.
- A1, A2, ...: The names of the exposure variables. As many “A” arguments should be supplied as the number of time points.
- L1, L2, ...: The names of the covariates at each time point. A vector of names should be supplied for each time point.
- A.fixed: For counterfactual functions, a matrix of dimension (number of regimes x timeobs), where each row represents a treatment regime ($\bar{A}$) for which counterfactual outcomes should be generated.
- distribution: For parametric functions, the names of the distribution of each variable in data, either “gaussian”, “binomial” or “multinomial”. If this is not supplied, the function tries to guess the distribution from the observed data.

The output is a list of nsim simulated datasets. For example, we used the following code to generate 1000 plasmode datasets in the nonparametric simulation scenario presented in the next section.
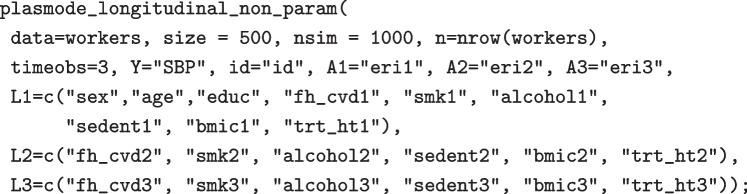


## Plasmode simulation study

In this section, we illustrate the use of our proposed plasmode algorithms by comparing estimators of the effect of the cumulative exposure to PSW on ABP. Data from 1,576 workers were used (“Data” section). We generated $J = 1000$ simulated datasets of size $m = 1000$ according to each of the plasmode algorithms. The true values of the coefficients were determined by our proposed counterfactual plasmode algorithms. The cumulative exposure effect was estimated in each simulated dataset using the methods described in “Marginal structural models and notation” section. When using IPTW, the standard error was estimated with a robust estimator, while for g-computation the standard error was estimated as the standard deviation of 50 nonparametric bootstrap replicates. We used this bootstrap approach rather than the percentile bootstrap to reduce the computation time. In the case of TMLE-SL, the standard error was estimated using the influence curve of the estimator. The 95% confidence intervals were obtained as the estimate $\pm 1.96$ the estimated standard error. The different estimating approaches were compared separately for the parametric and nonparametric plasmode algorithms. Methods were compared according to their bias (difference between the average of the coefficient estimates and the true value), the standard deviation of the estimates, the root mean squared error (RMSE), and the coverage of the 95% confidence intervals (proportion of the simulated datasets where the confidence interval included the true value).

Table 1 presents the results for the parametric plasmode scenario. Overall, we observed that estimates were more biased when using standard approaches. Among MSM estimators, the bias was lowest for g-computation with a sum of all biases of 0.56, compared to 0.77 for IPTW and 0.75 for both TMLE and TMLE-SL. Similarly, traditional methods had an overall larger RMSE than MSM estimators and g-computation had the lowest RMSE among MSM estimators. Almost all adjustment methods yielded a coverage of the confidence intervals fairly close to the nominal level of 95%. The Monte Carlo standard error was minimal: $\le 0.04$ for bias, $\le 0.03$ for standard deviation, and $\le$ 0.01 for coverage of confidence intervals [36].

**Table 1:** Comparison of the performance of different estimation methods of the effect of cumulative exposure to psychosocial stressors at work on blood pressure in the parametric plasmode simulation scenario

	Intermittent	Cessation	Onset	Chronic
**Bias**				
IPTW	-0.37	-0.03	0.23	-0.05
g-computation	0.27	0.02	0.16	-0.01
TMLE	0.26	-0.09	0.24	-0.07
TMLE+SL	0.25	-0.09	0.24	-0.07
Baseline adjustment	-0.40	-0.11	0.48	0.01
Baseline and Time-varying covariate adjustment	-0.44	-0.08	0.21	-0.19
**Standard deviation**				
IPTW	1.29	1.05	1.27	1.29
g-computation	0.53	0.84	0.86	1.05
TMLE	1.20	1.00	1.05	1.18
TMLE+SL	1.20	1.00	1.05	1.18
Baseline adjustment	1.12	0.98	1.06	1.17
Baseline and Time-varying covariate adjustment	1.11	0.98	1.06	1.19
**RMSE**				
IPTW	1.35	1.05	1.29	1.29
g-computation	0.59	0.84	0.88	1.05
TMLE	1.22	1.00	1.08	1.18
TMLE+SL	1.22	1.00	1.08	1.18
Baseline adjustment	1.19	0.98	1.16	1.17
Baseline and Time-varying covariate adjustment	1.20	0.98	1.08	1.21
**Coverage of the 95% confidence interval**				
IPTW	93%	96%	95%	95%
g-computation	90%	94%	94%	94%
TMLE	92%	94%	93%	93%
TMLE+SL	92%	94%	93%	93%
Baseline adjustment	92%	95%	92%	95%
Baseline and Time-varying covariate adjustment	92%	95%	94%	94%

Legend: IPTW = Inverse probability of treatment weighting, RMSE = root mean squared error, SL = Super Learner, TMLE = Targeted maximum likelihood

In Table 2, we present the results for the nonparametric plasmode scenario. Similar to the results obtained in the parametric plasmode scenario, standard approaches yielded estimates with a greater bias. The sum of all biases was lowest for IPTW with 1.79 compared with 2.02, 2.35 and 2.39 for g-computation TMLE and TMLE-SL, respectively. The standard deviations of the estimates for g-computation, TMLE, and TMLE-SL were smaller than those for IPTW or standard approaches. G-computation yielded the overall lowest RMSE. TMLE and TMLE-SL also had lower RMSE in general than either IPTW or standard approaches. Overall, standard approaches had poorer coverage of their confidence intervals than MSM estimators. All MSM estimators performed fairly similarly in terms of coverage of their 95% confidence intervals, with most values being in the 85%-95% range. The Monte Carlo standard error was $\le 0.05$ for bias, $\le 0.07$ for standard deviation, and $\le$ 0.02 for coverage of confidence intervals [36].

**Table 2:** Comparison of the performance of different estimation methods of the effect of cumulative exposure to psychosocial stressors at work on blood pressure in the nonparametric plasmode simulation scenario

	Intermittent	Cessation	Onset	Chronic
**Bias**				
IPTW	-0.51	0.04	-0.38	-0.34
g-computation	-0.59	-0.42	-0.39	-0.14
TMLE	-0.20	-0.75	-0.02	-0.91
TMLE+SL	-0.24	-0.73	-0.05	-0.90
Baseline adjustment	-0.94	-0.77	0.58	-0.90
Baseline and Time-varying covariates adjustment	-0.88	-0.82	0.04	-1.08
**Standard deviation**				
IPTW	1.02	1.49	1.16	2.93
g-computation	0.49	0.80	0.66	0.98
TMLE	0.90	0.84	0.63	1.43
TMLE+SL	0.90	0.84	0.63	1.42
Baseline adjustment	0.65	0.89	0.64	1.45
aseline and Time-varying covariates adjustment	0.65	0.85	0.70	1.50
**RMSE**				
IPTW	1.14	1.49	1.22	2.95
g-computation	0.77	0.90	0.77	0.99
TMLE	0.92	1.12	0.63	1.70
TMLE+SL	0.93	1.12	0.64	1.68
Baseline adjustment	1.14	1.15	0.90	1.75
Baseline and Time-varying covariates adjustment	1.09	1.21	0.65	1.81
**Coverage of the 95% confidence interval**				
IPTW	94%	91%	93%	71%
g-computation	73%	89%	89%	92%
TMLE	93%	83%	94%	86%
TMLE+SL	93%	83%	94%	87%
Baseline adjustment	70%	74%	80%	67%
Baseline and Time-varying covariates adjustment	71%	69%	93%	65%

Legend: IPTW = Inverse probability of treatment weighting, RMSE = root mean squarred error, SL = Super Learner, TMLE = Targeted maximum likelihood

## Discussion

We have introduced plasmode simulation algorithms adapted to settings with a time-varying exposure and time-varying covariates. As far as we know, such algorithms combining the advantage of artificial data and real-data had not been proposed for this setting before. The development of these algorithms was motivated by the objective of comparing traditional confounding adjustment methods with various estimators of the parameters of MSMs for the estimation of the effect of cumulative exposure to PSW.

The plasmode algorithms we have proposed have multiple strengths as compared to the standard approach to conducting simulation studies, which entails manually specifying all data-generating equations. First, our proposed plasmode algorithms allow generating realistic simulated data since the data-generating equations are determined from a real dataset. This feature also alleviates the need to manually specify multiple simulation parameters. In both plasmode and standard simulations, the true value of the parameter of interest can also be estimated by Monte Carlo simulation, for example by generating counterfactual outcomes when the parameter of interest is a causal effect. The main limitation of the plasmode algorithms as compared to fully synthetic simulations concerns their ability to investigate methods in various scenarios. Because the data-generating equations are dictated by the input data, all simulation scenarios based on the same data will inevitably have similar characteristics. Some of the only parameters that can be varied in our proposed plasmode algorithms are the sample size, which variables are considered in the algorithm, and whether parametric or nonparametric models are used to generate the simulated data. To some extent, this limitation can be mitigated by employing several input datasets. However, when the goal is to investigate the performance of statistical methods under specific conditions, employing standard simulation algorithms with manually specified parameters would be more appropriate. Using standard simulation algorithms allow varying many parameters between scenarios, such as the amount of collinearity between covariates, the probability distribution or density function of the variables being generated, the strength of the associations between generative and generated variables, and the exact functional form of the data-generating equations. Overall, standard and plasmode simulations thus have different and complementary roles in methods evaluation. Standard simulations are more suitable to evaluate methods in a variety of scenarios, whereas plasmode simulations simplify the process of evaluating methods in realistic scenarios.

A limitation of our longitudinal plasmode algorithms compared to point-exposure plasmode algorithms is that only the baseline covariates and baseline exposure are resampled from the original data, whereas the time-varying exposure, the time-varying covariates and the final outcome are all generated according to models fitted in the original data. The need to specify models for the time-varying exposure and covariates arise from the fact that the cumulative exposure effect depends on the exposure $\rightarrow$ confounders $\rightarrow$ outcome pathways. To be able to determine the true cumulative exposure effect, it must be possible to quantify these pathways, which is only possible if the data-generating equations for time-varying exposures, the time-varying confounders and the outcome are known. To mitigate this seemingly unavoidable limitation, we proposed determining the data-generating equations by fitting parametric or non-parametric models to the original data. As such, although models are used to generate most of the simulated data, instead of resampling, these models are made to share similarities with the original data.

The plasmode algorithms we have devised allowed us to compare empirically different methods for estimating the effect of cumulative exposure to PSW in context-specific realistic simulation scenarios. In the parametric plasmode simulation, traditional adjustment methods, wherein only baseline covariates or both baseline and time-varying covariates are included as covariates in an outcome model, had a slightly greater bias than MSM alternatives. In the more realistic and more challenging nonparametric plasmode scenario, the difference in bias between traditional and MSM methods was amplified, and traditional methods had undercovering confidence intervals, that is, confidence intervals that include the true exposure effect less often than expected. Among estimators of MSMs, g-computation overall produced the best results relative to bias, RMSE and coverage of confidence intervals. However, no method produced unbiased estimates with adequate coverage for all parameters in this nonparametric plasmode scenario. The nonparametric scenario likely reflects reality where parametric models are almost inevitably misspecified to some extent, leading to residual confounding bias. Our simulation suggests that this residual confounding due to model specifications would be of moderate size in the PSW context.

Our results provide empirical evidence supporting the importance of using MSMs when estimating the effect of cumulative exposure to PSW, since traditional adjustment approaches have been observed to have non-negligibly more bias than MSM approaches in our area-specific simulations. These results supplement the theoretical arguments that we had previously advanced in favor of using MSMs in this context [8]. However, we recognize that MSMs are more difficult to implement than conventional methods. While IPTW did not perform as well as g-computation, TMLE and TMLE-SL in our simulation study, it may represent a good compromise to improve the validity of analyses while remaining accessible to most analysts. It is also noteworthy that in our simulation study, the most complicated alternatives, TMLE and TMLE-SL, offered no particular benefit over the simpler g-computation approach. This may be because the machine learning algorithms we have considered within TMLE-SL were relatively simple. However, as mentioned previously, employing machine learning algorithms that are too flexible may also lead to invalid results [29, 30]. If employing flexible machine learning algorithms is necessary, using a cross-fitting technique can be required to ensure the validity of the TMLE-SL [29, 37]. A downside of employing such cross-fitting techniques is that it adds another layer of complexity to the analysis.

While the bias of traditional approaches was larger than that of MSM methods, it was relatively small as compared to what constitutes a meaningful difference in blood pressure from a clinical or public health perspective. As such, the impact of such bias on the conclusions of previous studies that used traditional approaches may be limited.

In conclusion, the plasmode algorithms we have developed can be important methodological tools for evaluating and comparing analytical methods for analyzing longitudinal data in realistic simulation scenarios. To facilitate the use of these algorithms, we provide R functions on a GitHub repository (https://github.com/detal9/LongitudinalPlasmode). From a substantive point of view, we recommend using MSMs when estimating the cumulative effect of PSWs.

## Data Availability

The data cannot be publicly shared because of constraints imposed by the Ethics Committee. Data can be privately shared after obtaining an approval from the Ethics Committee of the CHU de Québec – Université Laval. Please contact the corresponding author for more information.
